# Plasma lipoproteome in Alzheimer’s disease: a proof-of-concept study

**DOI:** 10.1186/s12014-018-9207-z

**Published:** 2018-09-20

**Authors:** Danni Li, Fangying Huang, Yingchun Zhao, Peter W. Villata, Timothy J. Griffin, Lin Zhang, Ling Li, Fang Yu

**Affiliations:** 1 Department of Laboratory Medicine and Pathology, University of Minnesota, 420 Delaware Street SE, MMC 609, Minneapolis, MN 55455 USA; 2 Masonic Cancer Center, University of Minnesota, Minneapolis, MN 55455 USA; 3 Department of Biochemistry, Molecular Biology, and Biophysics, University of Minnesota, Minneapolis, MN 55455 USA; 4 Department of Biostatistics, University of Minnesota, Minneapolis, MN 55455 USA; 5 Department of Experimental and Clinical Pharmacology, University of Minnesota, Minneapolis, MN 55455 USA; 6 School of Nursing, University of Minnesota, Minneapolis, MN 55455 USA

**Keywords:** Alzheimer’s disease, Plasma lipoproteins, Targeted proteomics, Complement C3, Very low-density lipoprotein (VLDL), Intermediate density lipoprotein (IDL), Low density lipoprotein (LDL), High-density lipoprotein (HDL), Biomarker, Diagnosis, Selected reaction monitoring (SRM)

## Abstract

**Background:**

Although total plasma lipoproteome consists of proteins that have shown promises as biomarkers that can identify Alzheimer’s disease (AD), effect sizes are modest. The objective of this study is to provide initial proof-of-concept that the plasma lipoproteome more likely differ between AD cases and controls when measured in individual plasma lipoprotein fractions than when measured as total in immunodepleted plasma.

**Methods:**

We first developed a targeted proteomics method based on selected reaction monitoring (SRM) and liquid chromatography and tandem mass spectrometry for measurement of 120 tryptic peptides from 79 proteins that are commonly present in plasma lipoproteins. Then in a proof-of concept case–control study of 5 AD cases and 5 sex- and age-matched controls, we applied the targeted proteomic method and performed relatively quantification of 120 tryptic peptides in plasma lipoprotein fractions (fractionated by sequential gradient ultracentrifugation) and in immunodepleted plasma (of albumin and IgG). Unadjusted p values from two-sample t-tests and overall fold change was used to evaluate a peptide relative difference between AD cases and controls, with lower p values (< 0.05) or greater fold differences (> 1.05 or < 0.95) suggestive of greater peptide/protein differences.

**Results:**

Within-day and between-days technical precisions (mean %CV [SD] of all SRM transitions) of the targeted proteomic method were 3.95% (2.65) and 9.31% (5.59), respectively. Between-days technical precisions (mean % CV [SD]) of the entire plasma lipoproteomic workflow including plasma lipoprotein fractionation was 27.90% (14.61). Ten tryptic peptides that belonged to 5 proteins in plasma lipoproteins had unadjusted p values < 0.05, compared to no peptides in immunodepleted plasma. Furthermore, 27, 32, 17, and 20 tryptic peptides in VLDL, IDL, LDL and HDL, demonstrated overall peptide fold differences > 1.05 or < 0.95, compared to only 6 tryptic peptides in immunodepleted plasma. The overall comparisons, therefore, suggested greater peptide/protein differences in plasma lipoproteome when measured in individual plasma lipoproteins than as total in immunodepleted plasma. Specifically, protein complement C3’s peptide IHWESASLLR, had unadjusted p values of 0.00007, 0.00012, and 0.0006 and overall 1.25, 1.17, 1.14-fold changes in VLDL, IDL, and LDL, respectively. After positive False Discovery Rate (pFDR) adjustment, the complement C3 peptide IHWESASLLR in VLDL remained statistically different (adjusted p value < 0.05).

**Discussion:**

The findings may warrant future studies to investigate plasma lipoproteome when measured in individual plasma lipoprotein fractions for AD diagnosis.

**Electronic supplementary material:**

The online version of this article (10.1186/s12014-018-9207-z) contains supplementary material, which is available to authorized users.

## Background

Tremendous work has taken place to identify AD biomarkers, especially using cerebrospinal fluid (CSF) and neuroimaging approaches [1, 2]. However, these CSF and neuroimaging biomarkers are appropriate for research purpose only and their use in a primary care clinical setting are limited due to safety concerns, costs, and requirements for specialized skills/facilities, which have and will continue to constraint the clinical use of these biomarkers. In contrast, blood-based biomarkers represent a clinically applicable alternative that would be both cost-effective and minimally invasive, ideal for wide clinical adoption [1, 2]. Plasma lipoproteome such as apolipoproteins E and J (Apo E and Apo J) play important roles in the pathophysiological development of AD and are promising blood-based biomarkers for early AD detection [3, 4]. Low levels of plasma Apo E and high levels of plasma Apo J have been associated with brain amyloidosis [4], hippocampal atrophy [4, 5], cognitive decline [6], and incident dementia (including AD) [7, 8]. However, effect sizes of the reported associations have been modest, which raises questions regarding their clinical significance in diagnosing AD [4, 7]. For instance, high plasma Apo J only increased AD diagnostic accuracy by 8% over age, sex, and *APOE* genotype alone [4]. To address this issue, a methodological approach to increase the diagnostic value of plasma lipoproteome is to discern individual plasma lipoprotein classes and fractionate plasma prior to analysis. This is because the total plasma lipoproteome are unevenly distributed across four major plasma lipoproteins (very low-density lipoprotein [VLDL], intermediate density lipoprotein [IDL], low density lipoprotein [LDL], and high-density lipoprotein [HDL]) [9, 10]. Discerning plasma lipoproteome in individual plasma lipoprotein classes may help uncover their unique relations with AD that would not otherwise be identifiable with total plasma lipoproteome.

The objectives of this proof-of-concept study are twofold. First is to establish a plasma lipoproteomic workflow that includes a targeted proteomic method based on selected reaction monitoring (SRM) and liquid chromatography and tandem mass spectrometry (LC/MS/MS) to perform relative quantitative peptide analyses in all 4 fractionated plasma lipoproteins (VLDL, IDL, LDL, HDL). Using a Fit-for Purpose approach, this targeted proteomic method is designed to be a “Tier 3” targeted peptide measurement, which is best suited for exploratory and discovery studies and only include limited isotope labelled reference peptides (not for every peptide) [11, 12]. More importantly, this study is designed to provide initial proof-of-concept that plasma lipoproteome more likely differs between AD cases (diagnosed based on clinical examination only) and controls when measured in individual plasma lipoprotein fractions than measured as total in immunodepleted plasma.

## Methods

### Reagents

ProteoPrep Immunoaffinity Albumin and IgG Depletion Kit was obtained from Sigma Aldrich (St. Louis, MO). All reagents were obtained from Thermo Fisher Scientific if not otherwise indicated. Water and acetonitrile (ACN) were Optima LC/MS grade. Isotopically labelled tryptic peptide standards DLLSPR (+ 6 Da mass shift for L, Fibrinogen), LGPLVEQGR (+ 6 Da mass shift for L and + 3 Da for G, ApoE), TYLPAVDEK (+ 6 Da mass shift for L, ApoC2), and YLYAR (+ 6 Da mass shift for L, SAA4) were synthesized locally in at the UMN (Dr. Laura Parker’s lab), and prepared in standard stocks with concentration of 1 mM in 20% ACN and 0.1% formic acid (FA). These four tryptic peptide standards were selected based from empirically obtained data. In the proteomic sample preparation described later, the peptide standards were added to trypsin digested peptides before desalting using stage tips that were made in-house using 200 μL pipette tips with 3 M (St. Paul, MN) Empore™ solid phase extraction disks styrenedivinylbenzene-reversed phase sulfonate (SDB-RPS) (Saint Paul, MN).

### Plasma samples

Ten fasting EDTA plasma samples were obtained from 5 AD cases and 5 age- and sex-matched controls. These 5 AD cases were from the FIT-AD Trial [13], in which AD dementia was diagnosed based on clinical evaluations only. These 5 controls were community dwelling subjects without dementia whose samples were purchased from the Solomon Park Research Laboratories and were requested to be processed in the same ways as samples of AD cases in the FIT-AD Trial. Details of blood collection and processing protocols are included in Additional file 1. All the plasma samples were stored at a − 80 °C freezer. This study was reviewed and approved by the University of Minnesota (UMN) Institutional Review Board.

### Fractionation of plasma lipoproteins

“Frozen never thawed” plasma aliquots were thawed and fractionated into 4 plasma lipoprotein classes (VLDL, IDL, LDL, and HDL) using a sequential gradient ultracentrifugation protocol with modifications [14]. Please see the protocol details in Additional file 1. All fractions were divided into small aliquots and stored at − 80 °C until further analysis.

### Immunoaffinity depletion

The aforementioned thawed plasma underwent albumin and IgG depletion by using the commercial ProteoPrep Immunoaffinity Albumin and IgG Depletion Kit per manufacturer’s instructions. Please see the protocol details in Additional file 1.

### Proteomics sample preparation

VLDL, IDL, LDL, HDL fractions and immunodepleted plasma samples (only depleted of albumin and IgG), each of 60 μL, underwent a delipidation protocol to remove lipids and extract proteins [15]. After delipidation, resulted protein pellets were re-suspended in a denaturing buffer (8 M urea and 0.4 M ammonia bicarbonate). Protein concentrations in the VLDL, IDL, LDL, HDL and plasma samples were determined using the BCA Protein Assay kit. The amount of proteins used for subsequent proteomics sample preparation for VLDL, IDL, LDL, HDL and plasma were 6.5, 3, 3, 10, and 10 μg, respectively. Protein samples were reduced using 10 mM TCEP (final concentration), alkylated using 12 mM iodoacetamide (final concentration), and trypsin digested (protein to trypsin mass ratio was 50:1) at 37 °C overnight. Trypsin digestion activity in the samples was stopped by freezing them for 20 min at − 80 °C. The four isotope labeled tryptic peptide standards were added to each sample (a final concentration of 50 nM per peptide in IDL, LDL, HDL, and immunodepleted plasma samples and of 67 nM in VLDL), which were dried down using speed vacuum for about 3 h. The resulted dried samples were reconstituted in a buffer of 2% ACN and 0.1% FA to reach the final concentration of 0.5 mg/mL (based on starting amount of proteins 10 μg and zero loss) and then desalted using a stage-tip protocol (Additional file 1).

### Targeted proteomics analysis based on SRM and LC/MS/MS

Two microliters of each of the desalted peptide samples were injected onto a home-packed analytical C18 reverse phase column (75 μm ID × 200 mm, 10 μm emitter orifice, Luna C18 5 μm particles [Phenomenex, Torrance, CA]). Peptides were eluted with buffer A (0.1% FA in water) and buffer B (0.1% FA in ACN) with the following gradient profile: 0–17 min, 2% B flow rate at 0.3 μL/min; 17–77 min, 2–45% B at 0.3 μL/min; 77–78 min, 45–90% B at 0.3 μL/min to 1 μL/min; 78–81 min, 90% B at 1 μL/min; 81–82 min, 90–2% B at 1 μL/min; 82–87 min, 2% B at 1 μL/min. Mass spectrometry detection was obtained on a TSQ Quantiva Triple Quadrupole (Thermo Scientific) in positive nanospray ionization mode. The mass spectrometry conditions were: spray voltage 2.0 kV, ion transfer tube temperature 350 °C, with collision energy in the range of 14.1 V to 42.2 V and a collision gas (argon) pressure of 1 mTorr. The resolution settings were 0.7 Da (full width at half-maximum) for both quadrupoles and transition dwell times were 10 ms.

### Peptide relative quantification

Skyline (version 3.6, 10493), an open source software, was used for quantitative data processing and proteomic analysis [16]. Specifically, Skyline was used to inspect peak integration and export integrated peak area along with other information such as background peak area. All integrated peaks were manually inspected to ensure correct peak detection, and integration was adjusted if necessary. To calculate relative peptide levels, background peak area was subtracted from total peak area of each SRM transition to calculate corrected peak area of all the SRM transitions including those for the peptide standards. Then, all the SRM transitions of the peptide standards in each sample were summed and used to normalize differences across all the samples due to variations in peptide analyses so that so the sums for every sample were equal. Then peak areas of 3 SRM transitions of a peptide was averaged to calculate its relative level.

### Statistical analysis

The relative level of each peptide was log-transformed (after adjustment of internal standards and batch effect if any), and two-sample t-tests were used to compare a peptide level between AD cases and controls. Fold changes were calculated as ratios of log-transformed relative level of a peptide between controls and AD cases. Furthermore, to account for multiple comparison testing, p values were adjusted using the method of positive False Discovery Rate (pFDR) [17]. Sample size and power considerations for future studies were also performed. Excel, R, and Graphpad Prism were used for data analysis.

## Results

### The targeted proteomics method

We developed a “Tier-3” targeted proteomic method [11] based on SRM and LC/MS/MS to perform relative quantitative analyses of 120 trypsin-digested peptides from 79 proteins present in plasma lipoproteins. This method included 4 isotope labeled peptides added after trypsin digestion to normalize variations in peptide analyses (e.g., desalting and LC/MS/MS analysis). To develop this targeted proteomic method, we had first performed shotgun proteomic experiments and identified plasma lipoproteins present in VLDL, IDL, LDL, and HDL. Then we used the shotgun proteomics data in combination the information available from the PeptideAltas spectral library to select proteins and tryptic peptides (i.e., trypsin enzyme only cleaves peptide chains at the carboxyl side of the amino acid lysine or arginine, except when either is followed by proline) which were subjected to Skyline for create a list of in silico SRM transitions. Briefly, 51 proteins were selected based on at least 2 tryptic peptide spectra available in our own spectral library (the shotgun proteomics data); and another 58 proteins were selected based on at least 1 tryptic peptide spectra available from our own spectral library (the rest tryptic peptides were selected from the PeptideAtlas spectral library to make 2–5 tryptic peptides per protein). Then, Skyline was used to select 5 transitions for each tryptic peptide. These in silico SRM transitions were further empirically tested using a sample mixed with equal amounts of tryptic peptides from VLDL, IDL, LDL, and HDL, which resulted in 79 proteins and 120 peptides, each protein with 1–3 signature peptide and each peptide of 3 SRM transitions. The selection of signature tryptic peptides was based on criteria reported previously [18–20]. Furthermore, we also empirically determined 3 SRM transitions for the 4 isotope labelled peptide standards. Additional file 2: Table S1 shows the list of 79 proteins and peptides selected for targeted analyses by SRMs, as well as precursor/fragment transition pairs and corresponding collision energies.

Within-day and between-days technical precisions of this targeted proteomic method (calculated as mean [SD] CVs % of all the 120 SRM transitions), evaluated by repeated measurement of a sample (mixed with equal weight of peptides from VLDL, IDL, LDL, and HDL) three times within a day (n = 3) and three times across three days (n = 3), were 3.95% (2.65) and 9.31% (5.59), respectively.

### Plasma lipoproteomics versus plasma proteomics

**Fig. 1:**
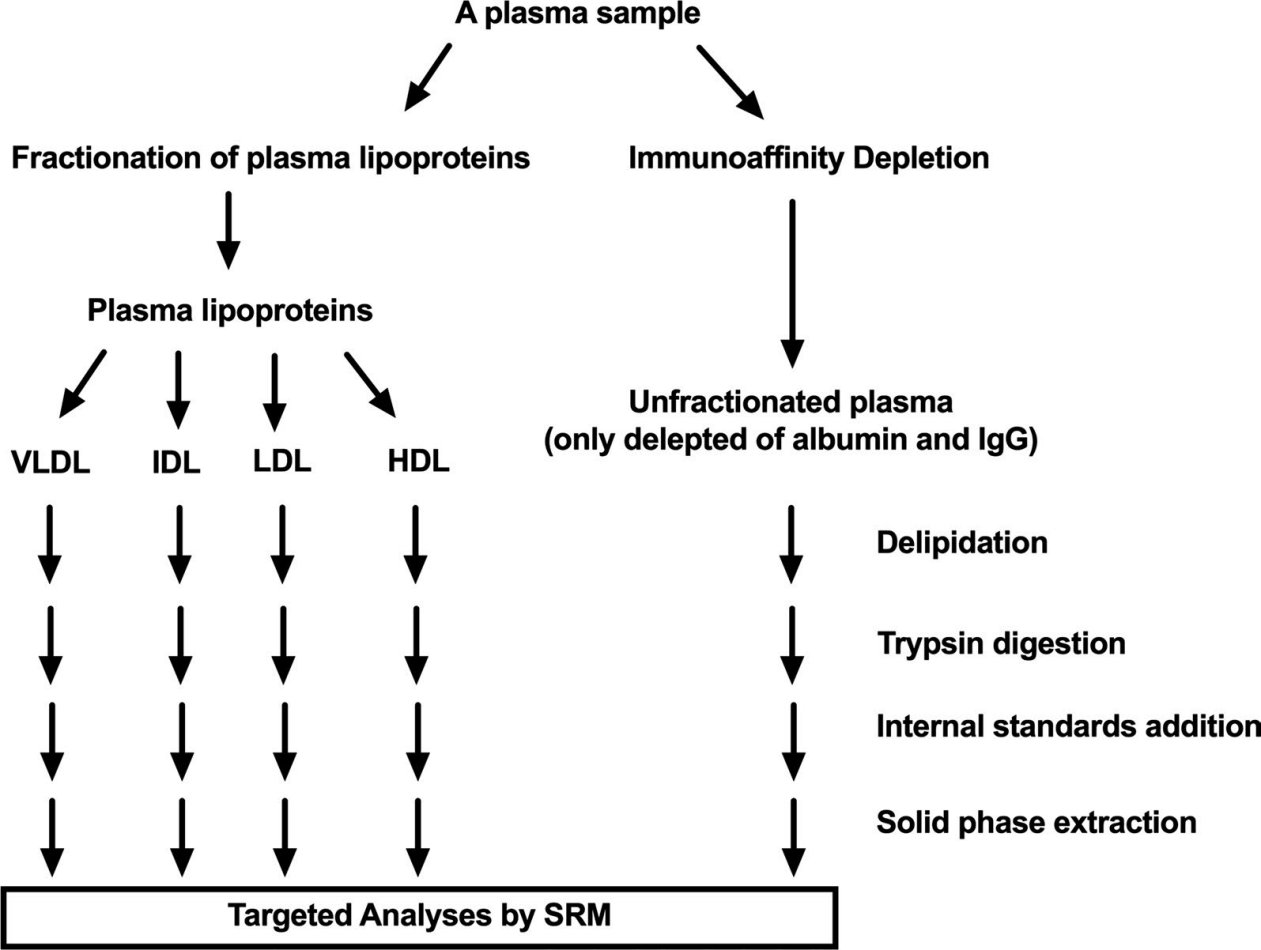
The study workflows

Figure 1 illustrates total and fractionated plasma lipoproteomic work flows that every plasma went through before analysis by the targeted proteomic method. The total plasma lipoproteomic work flow included plasma immunodepletion, a standard procedure that remove albumin and IgG, the two most abundance plasma proteins, which would otherwise interfere MS ability to detect total plasma lipoproteome. The fractionated plasma lipoproteomic workflow included fractionation of plasma lipoprotein by sequential ultracentrifugation, which was evaluated by processing three aliquots of a plasma sample across three days (one per day) and had an overall technical precision (mean [SD]) of 27.90% (14.61).

**Fig. 2:**
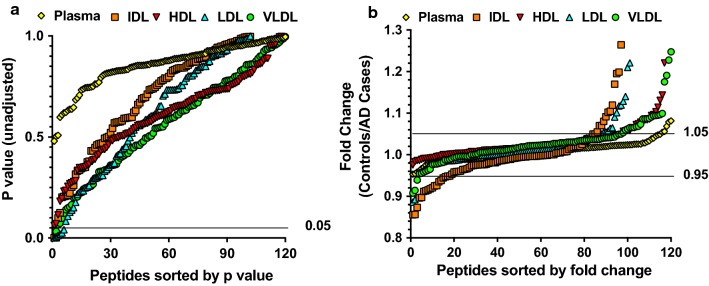
Comparison of unadjusted p values (**a**) and fold changes (**b**) for 120 trypsin-digested peptides (from 79 proteins) in VLDL, IDL, LDL, and HDL fractionated from plasma and in immunodepleted plasma obtained from AD cases (n = 5) and matched cognitively unimpaired controls (n = 5). Peptide levels were measured using MS-based targeted proteomics. Unadjusted p values were obtained from two-sample t-tests of peptide levels, and ratios of mean peptide levels in the controls to AD cases were used to indicate fold change

We then compared proteomics analysis results of fractionated plasma lipoproteins and immunodepleted plasma in a case–control study of 5 AD cases and 5 age and sex matched controls. The mean (SD) ages of the 5 AD cases and 5 controls were 80.8 (5.1) and 77.6 (3.6) years, respectively. Both the cases and controls included 4 males and 1 female (Table 1). Un-adjusted p values from two-sample t-tests and fold changes (calculated as ratios of mean relative level of a peptide between controls and AD cases) were used for the comparisons (Fig. 2a and b). None of the p values for the 120 peptides in immunodepleted plasma were lower than 0.05, compared to 2, 2, and 4 peptides in VLDL, IDL, and LDL had p values less than 0.05 (Table 2). Furthermore, 6 peptides of the fold changes for the 120 peptides in immunodepleted plasma had fold changes larger than 1.05 or less than 0.95, compared to 27, 32, 17, and 20 peptides in VLDL, IDL, LDL, and HDL. Since lower p values (< 0.05) and greater fold differences (> 1.05 or < 0.95) indicated stronger evidence of a difference between the cases and controls, the overall comparisons showed that greater protein differences were identified in plasma lipoproteins than in immunodepleted plasma.

**Table 1:** Demographics of the case and control subjects

Characteristic	Controls	Cases	p value
n	5	5	
Age (years)	80.8 ± 5.1	77.6 ± 3.6	0.28
Male (n)	4	4	1.00

**Table 2:** Top 10 proteins with the lowest unadjusted p values across all plasma lipoprotein fractions

Plasma lipoprotein fraction	Peptide	Protein	Unadjusted p value	FDR adjusted p value	Fold-changes (controls/AD cases)
VLDL	IHWESASLLR	C3	7.4987E−05	0.009073469	1.25
IDL	TLDPER	C3	0.00116714	0.059597026	1.19
IDL	IHWESASLLR	C3	0.00116857	0.059597026	1.17
LDL	IHWESASLLR	C3	0.00061261	0.062486488	1.14
LDL	TLDPER	C3	0.00678427	0.345997683	1.21
LDL	GNYDAAQR	Serum amyloid A4	0.01222158	0.415533787	1.04
LDL	ALSNVEGFER	Integrin alpha 2B	0.01827252	0.465949135	0.84
LDL	EALQGVGDMGR	Serum amyloid A4	0.02575496	0.52540128	1.06
LDL	VGYVSGWGR	Haptoglobin	0.04077834	0.693231851	1.1
VLDL	TTSGIHPK	chemokine (C-X-C motif) ligand 7	0.0324837	0.998795424	1.17

**Fig. 3:**
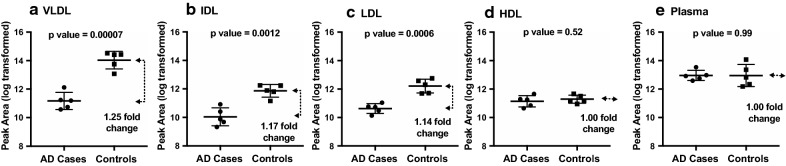
Comparison of complement C3 peptide IHWESASLLR levels in VLDL, IDL, LDL, and HDL fractionated from plasma and in immunodepleted plasma obtained from AD cases (n = 5) and matched cognitively unimpaired controls (n = 5). Peptide levels were measured using MS-based targeted proteomics. Unadjusted p values are displayed at the top of each plot, the middle lines indicate means, and the bottom and top lines indicate SD bars

Table 2 included the 10 peptides measured in VLDL, IDL or LDL with two-sample t-tests un-adjusted p values less than 0.05 and their corresponding fold changes. These 10 peptides belong to 5 proteins, including 5 peptides to complement C3. Figure 3 illustrated relative levels comparison of complement C3 peptide IHWESASLLR in VLDL, IDL, LDL, and HDL and in immunodepleted plasma. In contrast to no case-vs-control differences in IHWESASLLR when measured in HDL and in immunodepleted plasma (Fig. 3d, e), it had unadjusted p values of 0.00007, 0.00012, and 0.0006, respectively, and 1.25, 1.17, 1.14-fold changes, respectively, when measured in VLDL, IDL, and LDL (Fig. 3a, b, c). After pFDR adjustment, complement C3’s peptide IHWESASLLR in VLDL remained statistically significant difference between the cases and controls (FDR adjusted p value < 0.05). From the statistical analysis results, we estimated a standard deviation of 0.58 for the log-transformed complement C3 IHWESASLLR levels in VLDL. Based on the estimated standard deviation, we computed the powers for a two-sample *t* test to test the hypothesis of different means in log-transformed C3 IHWESASLLR levels in VLDL, for a planned future study with 30 cases and 30 controls. Table 3 presents the powers for a variety of values of effect size, with Bonferroni adjustment for six comparisons (assuming six protein biomarkers) and a family-wise Type I error rate of 0.05.

**Table 3:** Power of a future case control study at given effect sizes

N = 30/group				
Effect size	0.45	0.5	0.55	0.6
Power*	0.62	0.73	0.83	0.90

* At a significance level of 0.05, after adjustment for 6 comparisons (assumed number of protein biomarkers) using the conservative Bonferroni method

## Discussion

There have been studies using untargeted [9, 21] and targeted proteomics [22, 23] to investigate plasma lipoproteome in individual plasma lipoprotein fractions in cardiovascular, diabetes, and kidney diseases [10, 23–25]. To our knowledge, this study is the first to apply targeted proteomics to characterize plasma lipoproteome in AD and in plasma lipoprotein fractions beyond HDL. This study results suggest that the plasma lipoproteome when measured in plasma lipoprotein fractions (VLDL, IDL, LDL, and HDL) identifies more proteins with much stronger associations with AD when compared with analyses of total plasma lipoproteome in immunodepleted plasma.

This study especially observed complement C3 peptide IHWESASLLR’s difference between the cases and controls. Complement C3 is a protein of the immune system and plays a central role in the activation of complement system. Complement C3b adherence is a key step in the removal from the bloodstream of pathogens and proteins recognized as foreign. Previous studies have shown inconsistent associations between plasma complement C3 and AD. The majority of research demonstrated a higher plasma complement C3 level correlated with more brain amyloid burden [26, 27] and less hippocampal volume [28] in sporadic AD [29] and mutation carriers of autosomal dominant form of AD [30]. However, a recent study showed a lower plasma complement C3 level in amnestic mild cognitive impairment compared to normal [31]. This study indicated that the level of complement C3 was lower in VLDL, IDL, and LDL in AD cases than in controls. Because complement C3 mediated mechanisms seem to play important roles in the clearance of circulating amyloid beta [32], the findings on lower plasma VLDL, IDL and LDL complement C3 levels in AD may suggest the role of plasma lipoprotein associated C3 in Aβ clearance via complement mechanisms.

Despite these proof-of-concept results, this study has several limitations. First of all, it is limited by its small sample size. However, complement C3 IHWESASLLR in VLDL had pFDR adjusted statistically significant p value of 0.009 to distinguish the 5 AD cases and 5 controls. Second, the cases and controls were obtained from different cohorts and therefore there may be inherent bias. However, the bias, if it exists, would have affected both the immunodepleted and fractionated approaches equally. Third, we did not collect extensive demographic and genetic (e.g., *APOE* genotypes) information on these subjects and medications that might have affected plasma lipoproteins. Fourth, even though the entire workflow including fractionation of plasma lipoproteins using ultracentrifugation is well-established in literature and has been used to investigate plasma lipoproteome in cardiovascular disease [9, 10, 25], it is labor intensive. Future studies are needed to improve and automate the sample preparation workflow. Last but not the least, the AD cases were diagnosed based on clinical examination without amyloid deposition data. Because the context of use (COU) that we envision for proteins in plasma lipoproteins as biomarkers is to detect high likelihood of abnormal amyloid deposition in the brain of individuals who should be referred for amyloid neuroimaging to provide definitive measures (for either diagnostic or clinical trial enrollment purposes) [33], this proof-of-concept study results still need to be validated in a future case–control study of AD cases and controls with confirmed amyloid deposition data.

## Conclusions

This study provides initial evidence supporting a novel concept that measurement of plasma lipoproteome in plasma lipoprotein fractions may increase the accuracy of plasma lipoproteome in diagnosing AD. Future investigations including larger validation studies are needed to test plasma lipoproteome in plasma lipoprotein fractions as potential biomarkers in AD.

## Additional files

**Additional file 1**. Supplementary methods.

**Additional file 2: Table S1.** The list of proteins and peptides included in the targeted SRM analysis.
