# Supplementary material for: Plasma lipoproteome in Alzheimer’s disease: a proof-of-concept study
Source: Clin Proteomics. 2018 Sep 20;15:31. doi: 10.1186/s12014-018-9207-z (PMC6147047; doi:10.1186/s12014-018-9207-z)
Supplement: Supplementary file 1 — Additional file 1. Supplementary methods. [file 12014_2018_9207_MOESM1_ESM.docx]

**Supplementary Methods**

**Clinical Diagnosis of Alzheimer's Disease**

The FIT-AD Trial is a pilot randomized controlled trail to investigate the effects of a 6 month, individualized, moderate-intensity cycling interventions (20-50 minutes per session, 3 times a week) on cognition and hippocampal volume in community dwelling older adults with mild to moderate Alzheimer's disease (www.clinicaltrails.gov registration #: NCT1954550)[1]. AD diagnosis was made clinically following the NINCDS-ADRDA protocol [2]. The FIT-AD Trial inclusion and exclusion criteria are published [1].

**The FIT-AD Blood Collection and Processing Protocols for AD Samples**

Fasting blood samples were collected into BD EDTA tubes. These EDTA plasma tubes were gently mixed and centrifuged in 4°C at 3,000 g for 10 minutes. The tubes were then removed from the centrifuge immediately after completion. Plasma aliquots were made and the aliquoted samples were stored in the -80°C freezer. In this study, the AD plasma samples were stored no more than one year prior to biochemical analysis.

**Blood Collection and Processing Protocols for Control Samples**

Fasting EDTA plasma samples from control subjects without AD (age and sex matched to the AD samples) were purchased from the Solomon Park Research Laboratories and were requested to be processed in the same ways as those plasma samples in the FIT-AD Trial. Briefly, fasting blood plasma sample were collected into BD EDTA tubes, gently, mixed store on ice immediately. After centrifuge at 4 °C and 3000g for 10 minutes, plasma samples were aliquoted and store the specimens at -80°C freezer immediately after completion. In this study, the control plasma samples were stored no more than one year prior to biochemical analysis.

**Immunodepletion Protocol**

A plasma sample was diluted to 100 μL with the Equilibration Buffer, added to the top of the packed medium bed, and incubated at room temperature for 10 minutes. And the spin column and collection tube were then centrifuged at 8,000 g for 60 seconds. After centrifugation, the eluate in the collection tube was reapplied on the top of the medium bed and incubated for another 10 minutes at room temperature. After the incubation, the spin column and the collection tube were centrifuged for 60 seconds. The remaining unbound proteins were washed from the spin column by adding 125 μL of the Equilibration Buffer to the top of the medium bed and centrifuged for 60 seconds. The twice depleted plasma remained in the collection tube were combined with the wash from the previous step for optimal protein recovery. BCA protein assay was then used to measure protein concentration before the immunodepleted plasma used for further biochemical analysis. The depletion method is widely used and based on antibody affinity of albumin and IgG.

**Fractionation of Plasma Lipoproteins**

Briefly, potassium bromide (KBr) solutions were prepared in house with density verified by ASTM specific gravity hydrometers (VWR International, Radnor, PA) and stored at 4°C. Six hundred and forty microliters of plasma were pipetted into a polycarbonate tube and mixed well with 960μL of 1.006kg/L KBr (A) solution. After centrifugation at 15ºC with speed of 110,000rpm for 72 minutes (Optima MAX-XP Benchtop Ultracentrifuge with TLA 110 rotor, Beckham Coulter, Brea, CA), 640uL of supernatant layer was collected as VLDL. To the remaining volume, 38.9μL of 1.34kg/L (E) KBr and 601.1μL of 1.019kg/L (B) KBr solutions were added, and the resulted mixture was centrifuged at 15ºC with speed of 110,000rpm for 72 minutes. After removing top layer of liquid as IDL, 152.5μL of solution E and 487.5μL of 1.063kg/L (C) KBr were mixed with bottom layer of solution and centrifuged at 15ºC with speed of 110,000rpm for 90 minutes. The top layer was stored and labeled as LDL. The lower fraction was then mixed with 1085.6μL of solution E and 34.3μL of 1.21kg/L (D) KBr. After mixing well by pipette, 1.6mL of that solution was transferred to a clean polycarbonate tube and centrifuged at 15ºC with speed of 110,000rpm for 108 minutes. The HDL fraction is the top layer of the resulted solution.

**Desalting Stage-tip Protocol**

The stage tips were conditioned by washing through 60μL ACN and 60μL water consecutively. The sample dryness was reconstituted in 120μL of 0.2% FA in water and ensured the pH value is smaller than 3.0. The resulted sample solution was loaded into the stage tip and washed through the extraction membrane completely. Then the stage tips were washed twice by 120μL wash solvent A (95:5:0.2%, water: ACN: FA) and once by 60μL of ACN. Peptides were finally eluted from the extraction membrane by 60 µL elution solvent (60:35:5%, ACN: water: ammonium hydroxide). Following the stage-tip desalting, each sample was dried down again by speed vacuum. The dry pellets were then reconstituted by certain volume of 2%ACN and 0.1%FA buffer so that the concentration of protein/peptide is 0.5 mg/mL for subsequent targeted SRM analysis.

**References:**

**1.** Yu F, Bronas UG, Konety S, et al. Effects of aerobic exercise on cognition and hippocampal volume in Alzheimer's disease: study protocol of a randomized controlled trial (The FIT-AD trial). *Trials.* 2014;15:394.

**2.** McKhann G, Drachman D, Folstein M, Katzman R, Price D, Stadlan EM. Clinical diagnosis of Alzheimer's disease Report of the NINCDS‐ADRDA Work Group* under the auspices of Department of Health and Human Services Task Force on Alzheimer's Disease. *Neurology.* 1984;34(7):939-944.
